# Draft Genome Sequence of Paenibacillus profundus YoMME, a New Exoelectrogenic Gram-Positive Bacterium

**DOI:** 10.1128/mra.00235-22

**Published:** 2022-04-11

**Authors:** Yolina Hubenova, Eleonora Hubenova, Yordan Manasiev, Slavil Peykov, Mario Mitov

**Affiliations:** a Institute of Electrochemistry and Energy Systems, Bulgarian Academy of Science, Sofia, Bulgaria; b Department of Biochemistry and Microbiology, University of Plovdiv, Plovdiv, Bulgaria; c Medical Faculty, Rhenish Friedrich Wilhelm University of Bonn, Bonn, Germany; d Innovative Center for Eco Energy Technologies, Neofit Rilski South-West University, Blagoevgrad, Bulgaria; e Department of Genetics, Faculty of Biology, St. Kliment Ohridski Sofia University, Sofia, Bulgaria; f BioInfoTech Laboratory at Sofia Tech Park, Sofia, Bulgaria; University of Rochester School of Medicine and Dentistry

## Abstract

Paenibacillus profundus YoMME was isolated from the anodic biofilm of a sediment microbial fuel cell and recognized as one of the few exoelectrogenic Gram-positive bacteria, capable of transferring electrons extracellularly. Here, we report its draft genome sequence. The genome project is deposited at DDBJ/ENA/GenBank under the accession number JAJNBZ000000000.

## ANNOUNCEMENT

A new Gram-positive bacterium was isolated from the anodic biofilm of a microbial fuel cell, originally developed from freshwater sediment (1) from the Struma River, Bulgaria (global positioning system [GPS] coordinates, 41.990354, 23.067501), and recently identified as Paenibacillus profundus YoMME (2). This bacterium is a facultative anaerobe capable of forming electroactive biofilms and generating electricity (2).

The lack of information about its genome or similar annotated genomes in the NCBI genome database imposed the need for an in-depth genomic analysis.

The strain was stored, lyophilized, and cultured in liquid peptone meat broth, consisting of 10 g/L meat extract, 10 g/L Bacto peptone, and 5 g/L NaCl, pH 7.2, at 37°C on an orbital shaker at 120 rpm. Genomic DNA was extracted using the QIAamp DNA minikit (Qiagen) according to the manufacturer’s protocol. Whole-genome sequencing of *P. profundus* YoMME was performed using DNA nanoball sequencing technology. Genomic DNA (1 μg) was randomly size fragmented using a Covaris g-TUBE device, and fragments were size selected using magnetic beads to an average size of 200 to 400 bp. The purified fragments were end repaired, 3′-adenylated, ligated to adapters, and then PCR amplified. The final generated library had an insert size of 350 bp, and it was loaded onto an MGISEQ-2000 platform, where the sequencing step was performed using 2 × 150-bp paired-end reads (BGI Group, Hong Kong, China). All 4,328,631 resulting raw read pairs were next uploaded to the Galaxy online platform (3). Default parameters were used for all following software tools unless otherwise specified. The paired-end reads were first inspected using FastQC v0.11.9 (4); then, Trimmomatic v0.38 was used to remove adapters and discard sequences with a low Phred score (sliding window option, 4 bases; average quality required, 20) (5). Initial *de novo* assembly of the processed read pairs was carried out using the SPAdes v3.12.0 assembler with default settings (6). It generated a 6.92-Mb assembly (average genome coverage, 103×) comprising 93 contigs larger than 1,000 bp (largest contig, 671,236 bp), with an *N*_50_ value of 162,010 bp and an average GC content of 48.68%. Analysis using the CheckM v1.0.18 tool showed a level of completeness of 99.04% and 0% contamination. After receiving the draft genome sequence of *P. profundus* YoMME, a taxonomy analysis was performed using the Microbial Genomes Atlas Web server following the included workflow for the NCBI Genome Database, Prokaryotic section, with default settings (7). The closest matches by average amino acid identity (AAI) among all complete and chromosome-level genomes in the NCBI genome database are Paenibacillus alvei (GenBank accession number LS992241; 69.79% AAI) and Paenibacillus thiaminolyticus (CP041405; 68.68% AAI). Next, the genome of *P. profundus* YoMME was annotated using the PGAP (8). The genome consists of 6,327 genes, 6,261 coding sequences (CDSs), 6 rRNAs, and 55 tRNAs and most likely belongs to a species that is not represented in the database (*P* = 0.0025).

The established and annotated genome sequence of *P. profundus* YoMME will be a reference point for elucidating the mechanisms of extracellular electron transfer and the bacterial components involved.

### Data availability.

The whole-genome shotgun project for *P. profundus* YoMME has been deposited at DDBJ/ENA/GenBank under the accession number JAJNBZ000000000. The version described in this paper is version JAJNBZ000000000.1. The raw sequencing data are available in the SRA under the accession number SRP354977.
